# Chromosome-level analysis of the *Colletotrichum graminicola* genome reveals the unique characteristics of core and minichromosomes

**DOI:** 10.3389/fmicb.2023.1129319

**Published:** 2023-03-23

**Authors:** Sioly Becerra, Riccardo Baroncelli, Thaís R. Boufleur, Serenella A. Sukno, Michael R. Thon

**Affiliations:** ^1^Department of Microbiology and Genetics, Institute for Agrobiotechnology Research (CIALE), University of Salamanca, Villamayor, Spain; ^2^Department of Agricultural and Food Sciences (DISTAL), University of Bologna, Bologna, Italy; ^3^Department of Plant Pathology and Nematology, Luiz de Queiroz College of Agriculture, University of São Paulo, Piracicaba, Brazil

**Keywords:** plant pathogenic fungus, hybrid assembly, repetitive DNA, repeat-induced point mutation (RIP), dispensable chromosomes

## Abstract

The fungal pathogen *Colletotrichum graminicola* causes the anthracnose of maize (*Zea mays*) and is responsible for significant yield losses worldwide. The genome of *C. graminicola* was sequenced in 2012 using Sanger sequencing, 454 pyrosequencing, and an optical map to obtain an assembly of 13 pseudochromosomes. We re-sequenced the genome using a combination of short-read (Illumina) and long-read (PacBio) technologies to obtain a chromosome-level assembly. The new version of the genome sequence has 13 chromosomes with a total length of 57.43 Mb. We detected 66 (23.62 Mb) structural rearrangements in the new assembly with respect to the previous version, consisting of 61 (21.98 Mb) translocations, 1 (1.41 Mb) inversion, and 4 (221 Kb) duplications. We annotated the genome and obtained 15,118 predicted genes and 3,614 new gene models compared to the previous version of the assembly. We show that 25.88% of the new assembly is composed of repetitive DNA elements (13.68% more than the previous assembly version), which are mostly found in gene-sparse regions. We describe genomic compartmentalization consisting of repeat-rich and gene-poor regions vs. repeat-poor and gene-rich regions. A total of 1,140 secreted proteins were found mainly in repeat-rich regions. We also found that ~75% of the three smallest chromosomes (minichromosomes, between 730 and 551 Kb) are strongly affected by repeat-induced point mutation (RIP) compared with 28% of the larger chromosomes. The gene content of the minichromosomes (MCs) comprises 121 genes, of which 83.6% are hypothetical proteins with no predicted function, while the mean percentage of Chr1–Chr10 is 36.5%. No predicted secreted proteins are present in the MCs. Interestingly, only 2% of the genes in Chr11 have homologs in other strains of *C. graminicola*, while Chr12 and 13 have 58 and 57%, respectively, raising the question as to whether Chrs12 and 13 are dispensable. The core chromosomes (Chr1–Chr10) are very different with respect to the MCs (Chr11–Chr13) in terms of the content and sequence features. We hypothesize that the higher density of repetitive elements and RIPs in the MCs may be linked to the adaptation and/or host co-evolution of this pathogenic fungus.

## Introduction

*Colletotrichum* is a genus of filamentous fungi and one of the most common and destructive groups of plant pathogens, causing disease in plants from nearly every crop and natural ecosystem worldwide and resulting in substantial economic losses (Prusky et al., 2000; Dean et al., 2012; Baroncelli et al., 2017). Among important potential hosts are cereals and legume crops such as maize and soybean and important fruits such as olives and strawberries (Talhinhas et al., 2005; Frey et al., 2011; Baroncelli et al., 2015; Boufleur et al., 2021). The genus contains ~250 species organized into 15 main phylogenetic lineages, which are known as species complexes (s.c.) (Baroncelli et al., 2017; Samarakoon, 2018; Damm et al., 2019; Talhinhas and Baroncelli, 2021). *Colletotrichum* fungi are also important as experimental models in studies of many aspects of plant disease (Baroncelli et al., 2016). In the 1970s, *C. graminicola* caused extensive epidemics of maize anthracnose in the United States, leading it to become a model pathogen for research in plant pathology.

Maize (*Zea mays*) is one of the most important crops worldwide (Wu and Guclu, 2013). With a harvested area of more than 192 million hectares, maize is now the second most extensively cultivated cereal crop (FAOSTAT, 2016). Leaf blight and stalk rot of maize (Bergstrom and Nicholson, 1999) are important maize diseases, producing annual yield losses of more than one billion dollars in the United States alone (Frey et al., 2011). The pathogen can infect all plant parts and can be found throughout the growing season.

The first version of the *C. graminicola* genome was published in 2012, providing a significant resource to the scientific community (O'Connell et al., 2012). In the past 10 years, DNA sequencing technology has advanced considerably. Higher resolution and more complete genome sequence assemblies are now possible, although, in eukaryotic genomes, it is also possible to find some fragmented genomes when only one sequencing technology is used (Faino et al., 2015). Nowadays, third-generation sequencing technologies such as that provided by the PacBio platform (Pacific Biosciences) can achieve more complete scaffolds, often arriving at complete chromosome sequences (Schadt et al., 2010; Liu et al., 2012). Although it has a higher error rate than second-generation sequencing, the errors can be corrected by combining high-fidelity short-read sequences from platforms such as those provided by Illumina Inc. with longer reads, generating a hybrid genome assembly (Rhoads and Au, 2015). In addition, long-reads can improve the assembly of repeat-rich genomic regions often found in pathogenic fungi and provide important information on genome structure and evolution (Möller and Stukenbrock, 2017).

The genome of *C. graminicola* M1.001 (V1) was assembled using Sanger sequencing, 454 pyrosequencing, and an optical map to obtain an assembly of 13 pseudochromosomes (O'Connell et al., 2012). A cytological analysis verified that the genome contains 13 chromosomes, the smallest of which are referred to as minichromosomes (MCs) (Taga et al., 2015). MCs can be differentiated from other chromosomes in plant pathogenic fungi by their unusually small size (Griffith, 1975; O'Sullivan et al., 1998). In *C. graminicola* and *C. higginsianum*, the MCs are <1 Mb (O'Connell et al., 2012; Dallery et al., 2017), but the size and number of MCs may vary even between closely related species (Gan et al., 2021) and among isolates of the same species (Zolan, 1995; Orbach, 1996; Covert, 1998; Pires et al., 2016). Not all members of *Colletotrichum* contain MCs. For example, several species, such as *C. orbiculare*, lack them, or at least they have not been described yet (Taga et al., 2015).

Some authors define MCs as dispensable chromosomes as they are missing in some strains and are not required for normal physiological functions (Miao et al., 1991; Masel, 1996). In some cases, they appear to harbor pathogenicity genes and play an important role in pathogenic adaptation, and can be absent in non-virulent strains (Ma et al., 2010). Moreover, MCs can have different functions in the same species. For example, in *C. higginsianum*, one MC is related to virulence, while the other affects neither virulence nor growth *in vitro* (Plaumann et al., 2018). The MCs of plant pathogenic fungi may carry genes encoding effectors, molecules that aid the colonization of the host cell by modulating the plant's immune system (Win et al., 2012; Balesdent et al., 2013; Dallery et al., 2017; Bhadauria et al., 2019; Peng et al., 2019). Species that lack MCs, such as *C. orbiculare*, can have genomic compartments that are gene-sparse and enriched with repetitive DNA sequences as well as gene-rich regions that have few repetitive sequences (Gan et al., 2013; Dong et al., 2015).

We present a new genome assembly of *C. graminicola* strain M1.001 using a combination of PacBio and Illumina sequencing technologies. This hybrid method allowed the assembly of a genome sequence with 13 chromosomes, of which eight are assembled telomere to telomere, including three MCs, of which Chr11 and Chr13 also have telomeric repeats at both ends. We found characteristics of compartmentalization in the genome, with higher repeat content in the MCs which lack secreted proteins and, therefore, effectors. The core chromosomes (Chr1–Chr10) are very different with respect to the MCs (Chr11–Chr13) in terms of the content and sequence features. We hypothesize that the higher density of repetitive elements and RIPs in the MCs may be linked to the adaptation and/or host co-evolution of this pathogenic fungus.

## Materials and methods

### Fungal strains and DNA extraction

In this study, we utilized *C. graminicola* strain M1.001. This strain was obtained from symptomatic maize plants during a survey of *Colletotrichum* spp. associated with anthracnose (Vaillancourt and Hanau, 1991) in Missouri in 1978. Total DNA was extracted from 3-days-old *C. graminicola* colonies grown in potato dextrose broth (PDB) under agitation (150 rpm) at 25°C (Sanz-Martín et al., 2016). The mycelium was vacuum filtered, immediately frozen in liquid nitrogen, and stored at −80°C until the DNA extraction.

The mycelium was macerated with liquid nitrogen using a mortar and pestle. One-third of the 1.5 mL Eppendorf tube was filled with powdered mycelium, and high molecular weight (HMW) DNA was extracted following a modified cetyltrimethylammonium bromide (CTAB) protocol for fungal genomic DNA (Murray and Thompson, 1980; Baek and Kenerley, 1998; Irfan et al., 2013). DNA was quantified by fluorometry (Qubit), and the DNA Integrity Number (DIN) was determined with one ScreenTape (5067-5365) of the Genomic DNA kit Agilent on the 2200 TapeStation system.

### DNA sequencing, genome assembly, and quality

The *C. graminicola* genome was sequenced using a combination of short- and long-reads. Long-reads were generated using PacBio RS Single Molecule Real-Time (SMRT) (Pacific Biosciences) with one Sequel II SMRT cell. Short-read sequencing was performed on a NovaSeq 6000 using a 151 bp paired-end library. Both services were performed by The Center d'Expertise et de Services (Genome Québec CES, Canada).

The quality of the sequenced reads was checked with FastQC v.0.11.9 for Illumina, and with LongQC v.1.2.0.b for PacBio (Fukasawa et al., 2020). Low-quality reads and adaptors were trimmed with Trim Galore v.0.6.4. Short-reads were merged with Flash v.1.2.7 (Magoc and Salzberg, 2011). The hybrid assembly was divided into four steps: assembly, polishing, synteny analysis, and the last step of final polishing.

PacBio raw data was assembled with Canu v.2.1.1 (Koren et al., 2017) with parameter genome size = 55 m. Pilon v.1.23 (Walker et al., 2014) was used to polish the draft assembly, by aligning the short-reads with Bowtie2 v.2.3.5.1 (Langmead and Salzberg, 2012). The files were converted and sorted with samtools v.1.10 (Li et al., 2009).

To assign contigs to chromosomes, a synteny analysis was performed between the draft assembly and V1 assembly using SyMap v.5.1.0 (Soderlund et al., 2006). The syntenic contigs were concatenated by entering 100 Ns corresponding to the gaps. To assure these contigs correspond to the same one for each concatenated sequence, three rounds of Pilon v.1.23 with the Illumina data were used. Gaps were filled by changing the Ns for the corresponding nucleotide. Five iterations of Pilon v.1.23 were used to obtain the final version of the assembly (V4). V2 and V3 were internal versions that were not released to the public.

The genome quality was assessed using QUAST (Gurevich et al., 2013) web interface by CAB (Center for Algorithmic Biotechnology), and the completeness by using BUSCO v.5.2.2 (Manni et al., 2021) lineage dataset—sordariomycetes_odb10. The mitochondrial genome was obtained in a contig and verified by Blast from NCBI. The structural rearrangements of the V1 to V4 genome assemblies of *C. graminicola* M1.001 were checked out using plotsr v.0.4.1 (Goel and Schneeberger, 2022).

### Repetitive DNA annotation

To determine repetitive DNA elements, we used the Software RepeatModeler v.2.0.3 (Flynn et al., 2020) with LTRStruct, using RECON v.1.08 (Bao and Eddy, 2002), Repeat Scout v.1.0.6 (Price et al., 2005), and LtrHarvest v.1.5.9 (Ellinghaus et al., 2008)/Ltr_retriever v.2.6 (Ou and Jiang, 2018). The assembly was masked using RepeatMasker v.4.0.7 with the consensus libraries. We used RIPper (van Wyk et al., 2019) to identify the regions affected by RIP, and the following suggested parameters were used: a window size of 1Kb and slide size of 500 bp, 0.01 minimum composite, 1.1 minimum product, and maximum substrate of 0.75. This pipeline was applied for the other assemblies included in this research to obtain the same parameters that were then analyzed.

### Gene prediction and functional annotation

Gene prediction was performed using all the raw RNA-Seq reads of *C. graminicola* M1.001 available in the MycoCosm database (https://mycocosm.jgi.doe.gov/) and the Sequence Read Archive (https://www.ncbi.nlm.nih.gov/sra) as biological evidence. The rnaSPAdes (Bushmanova et al., 2019) tool of SPAdes v.3.11.1 (Prjibelski et al., 2020) was used to generate.fasta transcripts files. The transcripts were assembled into the draft assembly (V4), which was considered as the input transcripts in the MAKER pipeline. By mapping the RNA-Seq reads to our draft assembly, we obtained 62,289 sequences. We downloaded 135,818 proteins with “like-protein” homology, from the genus *Colletotrichum* from JGI (DOE Joint Genome Institute, 2021) Mycocosm (Grigoriev et al., 2014). At the same time, the web interface of Training Augustus (http://bioinf.uni-greifswald.de/webaugustus/training) (Stanke et al., 2008) was used with the draft assembly (V4) as a genome file, and the transcripts file was obtained from *de novo* transcriptome assembly as the cDNA file.

MAKER v.3.01.03 (Cantarel et al., 2008) was adjusted to make a final selection from GeneMark (Besemer and Borodovsky, 2005), Augustus v.3.3.3, and SNAP to predict the *ab initio* gene. GeneMark was used with the parameters –ES, –fungus, and –sequence. The assembled RNA-seq was also used to run the MAKER pipeline. A consensus repeat library was obtained from RepeatMasker v.4.0.7. The.ctl files were designed using the proteins, transcripts, repeat masking, and GeneMark gene prediction, and then we ran MAKER to select the best prediction. A total of 15 genes were manually modified, of which eight incomplete genes were discarded, and seven were corrected. We used the MITOS web server (Bernt et al., 2013) to annotate the mitochondrial genome.

We broadly defined candidate effectors as secreted proteins and we first used SignalP v.5.0b (Almagro Armenteros et al., 2019b) to predict the presence of a signal peptide. We then used TargetP 2.0 (Almagro Armenteros et al., 2019a) to confirm that the proteins were not targeted to organelles. Next, we used PredGPI (Pierleoni et al., 2008) to assure that the proteins were not predicted to have GPIanchors, and filtered out the proteins with transmembrane domains using TMHMM (Sonnhammer et al., 1998). These proteins we refer to as extracellular secreted proteins (SP). Proteins with the presence of a signal peptide were used as input on Effector-P3 (Sperschneider and Dodds, 2022), to classify them as predicted effectors.

### Analysis of gene and repeat distribution

The average distance from genes, secreted proteins, and predicted effectors to the closest repeat was determined with BEDtools v.2.27.1 (Quinlan and Hall, 2010) using the “closest” script with -d and -io parameters. The.bed files generated were used to produce violin plots using the R package ggplot2 and the function geom_violin. The distance between SP and predicted effectors and the nearest repeat was compared to the distance between all genes and the nearest repeat using the permutation-based test contained in the R package regioneR (Gel et al., 2016). We performed 10,000 permutations, following the approach reported by Dallery et al. (2017). To determine the distribution and content in each chromosome by size, principal component analysis was performed using the percentage of genes, secreted proteins (SP), predicted effectors, hypothetical proteins (HP), RIPs, and repeats, by plotting the principal component analysis from RStudio v.4.0.2 with “prcomp” and visualizing it with biplot.

The core and dispensable percentage of genes for each chromosome was determined for the 21 strains of *C. graminicola* reported by Rogério et al. (2022). Proteins were clustered based on their similarity with OrthoFinder v.2.5.4 (Emms and Kelly, 2019). We then verified the gene content with transcripts using BEDtools v.2.27.1 (Quinlan and Hall, 2010) with the script “intersect”. Genes associated with the MCs were checked by orthogroup using Geneious Blastp. Genes with ≥90% of coverage and ≥60% of identity were considered similar (Boufleur et al., 2021).

### Comparative assembly analysis with other assemblies of *Colletotrichum*

Seven genomes of *Colletotrichum* available at NCBI were selected for downstream analysis (Table 1). A phylogenomic tree was constructed with all proteomes, using *Verticillium dahliae* as an outgroup. The proteins were clustered with Orthofinder v.2.5.4 (Emms and Kelly, 2019), and 5,348 single copy (per genome) orthogroups were aligned with MAFFT v.7.453 (Katoh and Standley, 2013). Gblocks v.0.91b (Talavera and Castresana, 2007) was used to detect and remove low-quality alignments. The phylogenetic tree was built with FastTree v.2.1.11 Double precision (No SSE3) (Price et al., 2010). QUAST, BUSCO v.5.2.2, RepeatModeler v.2.0.3, RepeatMasker v.4.0.7, and RIPper were also used for genome characterization.

**Table 1:** Genome assemblies sequenced with long-read technology of the *Colletotrichum* species used in this study.

**Species**	**Strain**	**Species complex**	**Assembly size (Mb)**	**#Sequences**	**#Predicted genes**	BUSCOs^*^	**Accession number**
*C. siamense*	Cg363	Gloeosporioides	62.94	22	15,190	C: 98.3% (S: 97.9%, D: 0.4%) F: 0.5%, M: 1.2%	GCA_013390195.1
*C. fructicola*	Cf413	Gloeosporioides	56.50	12	15,647	C: 98.4% (S: 98.2 %, D: 0.2%) F: 0.3%, M: 1.3%	GCA_013390205.1
*C. fructicola*	Nara gc5	Gloeosporioides	59.54	12	17,388	C :98.4% (S: 98.2%, D: 0.2%) F: 0.3%, M: 1.3%	GCA_000319635.2
*C. lupini*	RB221	Acutatum	63.41	11	18,324	C: 98.2% (S:98.0%, D: 0.2%) F: 0.6%, M: 1.2%	GCA_023278565.1
*C. scovillei*	TJNH1	Acutatum	52.00	15	13,417	C: 98.1% (S: 97.6%, D: 0.5%) F: 0.7%, M: 1.2%	GCA_011075155.1
*C. higginsianum*	IMI 349063	Destructivum	45.95	11	13,330	C: 89.8% (S: 89.5%, D: 0.3%) F: 0.4 %, M: 9.8%	GCA_001672515.1

* Complete BUSCOs (C) | Complete and single-copy BUSCOs (S) | Complete and duplicated BUSCOs (D) | Fragmented BUSCOs (F) | Missing BUSCOs (M). The # symbol indicates “Number of”.

## Results

### Improved *C. graminicola* assembly reveals 13 predicted chromosomes

PacBio sequencing yielded 7,331,198 reads (138 Gb) using one Sequel II SMRT cell. The longest read was 212,531 bp and the mean read length was 10,044 bp. The reads were assembled with Canu into 22 contigs with a total length of 57,627,041 bp. Illumina sequencing yielded 87,754,330 reads. After five rounds of polishing with Illumina data, we obtained an assembly of 57,626,709 bp in length comprised of 22 contigs. We then manually checked the composition of the contigs using blast searches and identified three contigs (134,681 bp) with bacterial segments which were removed. One contig was identified as the mitochondrial genome and was removed for separate analysis.

We performed an analysis of synteny between the 13 pseudochromosomes of the previous version of the genome (O'Connell et al., 2012) and the 18 contigs resulting from our draft assembly. Five contigs were obtained by concatenating the syntenic contigs together. After three rounds of polishing, the assembly contained 13 contigs, which is the matching number for a complete chromosome-level assembly. The total size of the V4 assembly was 57.43 Mb (Tables 2, 3) with a mean coverage of 314X, and it was 15% longer than V1.

**Table 2:** Summary of assembly statistics for the *C. graminicola* M1.001 genome.

**Description**	**V1**	**V4**
#Contigs	654	13
Size (Mb)	51.64	57.43
N50	579,194	6,444,216
L50	27	5
GC (%)	49.12	45.98
Ns	0	181
Ns per-100 kbp	0	0.315
Complete BUSCOs (%)	C:97.3% (S:97.1%, D:0.2%)	C:98.2% (S:97.9%, D:0.3%)
Fragmented (%)	0.7	0.6
Missing BUSCOs (%)	2.0	1.2

The # symbol indicates “Number of”.

**Table 3:** Comparison of *C. graminicola* M1.001 assembly versions by chromosome.

	**V1 (2012)**			**V4 (2022)**		
**Chr**	**Size (bp)**	**5**′**Telomere**	**3**′**Telomere**	**Size (bp)**	**5**′**Telomere**	**3**′**Telomere**
1	6,787,984	NA	NA	7,652,868	Yes	Yes
2	6,748,533	NA	NA	7,519,183	NA	Yes
3	6,027,927	NA	NA	6,761,645	Yes	Yes
4	5,882,731	NA	NA	6,472,013	Yes	NA
5	5,174,327	NA	NA	6,444,216	Yes	Yes
6	4,553,816	NA	NA	5,007,504	Yes	Yes
7	4,372,745	NA	NA	4,680,494	Yes	Yes
8	2,496,009	NA	Yes	4,148,198	Yes	Yes
9	3,032,982	NA	NA	3,633,823	Yes	NA
10	3,325,116	NA	NA	3,267,738	NA	NA
11	289,653	NA	NA	729,294	Yes	Yes
12	169,361	NA	NA	559,114	Yes	NA
13	138,786	NA	NA	550,543	Yes	Yes

**Figure 1:**
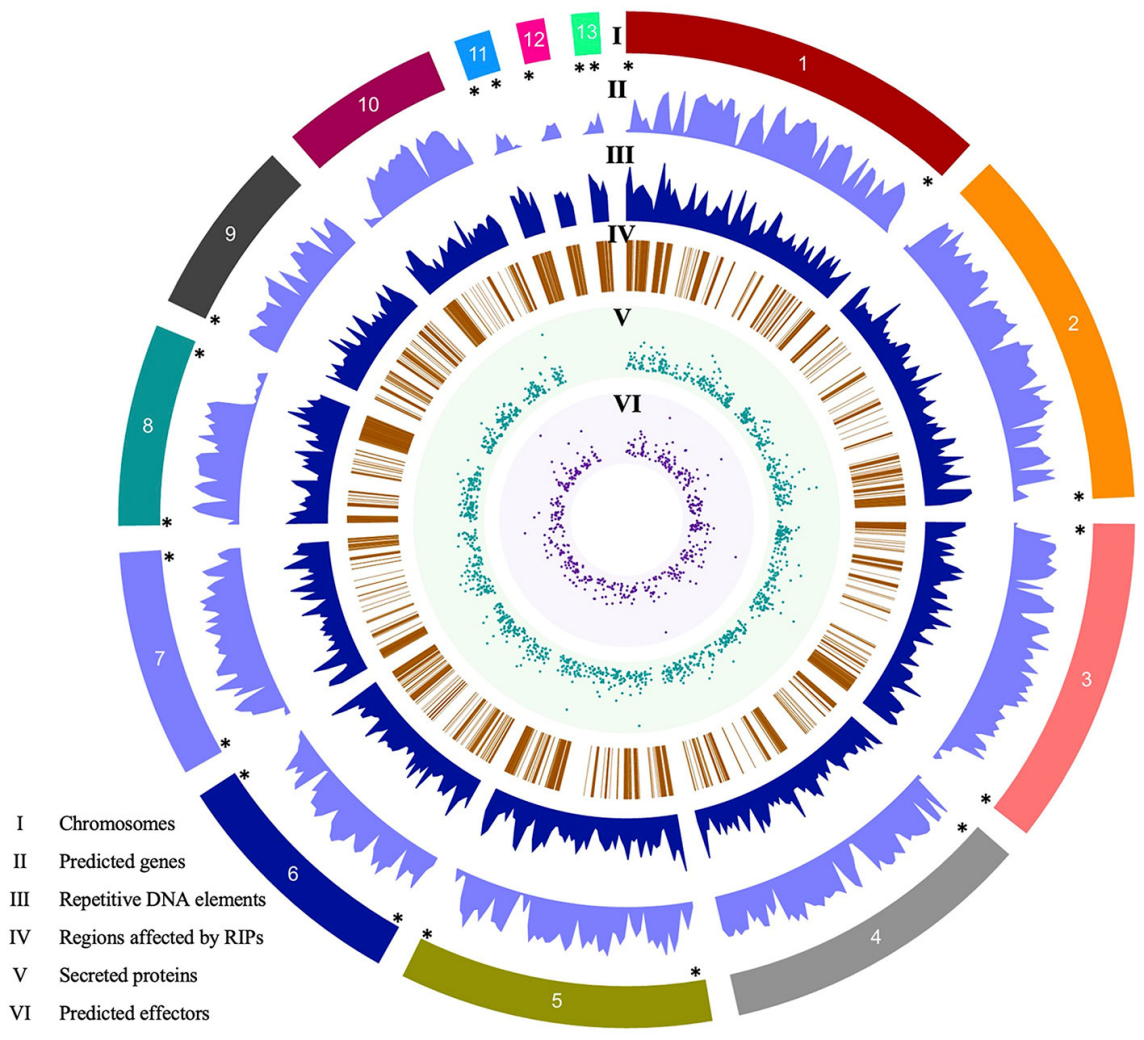
Circos plot of the *Colletotrichum graminicola* M1.001 V4 assembly created with Circa. Tracks: (I) 13 chromosomes, (II) number of predicted genes, (III) repetitive DNA elements, (IV) regions affected by RIPs mutations, (V) number of secreted proteins predicted with SignalP5, (VI) number of candidate effectors predicted by EffectorP3. (*) Shows eight complete chromosomes (Chr1, Chr3, Chr5–Chr8, Chr11, and Chr13), Chr4, Chr9, and Chr12 with the 5′ telomeric region, Chr2 with 3′ telomeric regions, and the Chr10 lacks a telomeric region. Genes are inversely distributed with respect to repeats (tracks II and III). RIPs mutations are also more frequent in gene-poor regions. Chromosomes 11, 12, and 13 lack secreted proteins and therefore predicted effectors.

Eight contigs are complete chromosomes (Chr), terminating in telomeric repeats (TTAGGG)_n_, at both ends. Contigs 2, 4, 9, and 12 have one end comprised of telomeric repeats, while contig 10 lacks telomeric repeats. The contigs are called chromosomes. Three chromosomes (Chr11, Chr12, and Chr13) were identified as MCs due to their size and similarity to the three reported by O'Connell et al. (2012). MCs, Chr11, and Chr13, contain both telomeric regions, while Chr12 contains one telomeric region (Figure 1). The mitochondrial genome of the V4 assembly is a single contig, 67,326 bp in length, and is nearly twice as long as the mitochondrial genome of the V1 assembly (39,649 bp). Further investigation revealed that a contig representing a portion of the mitochondrial genome of V1 was included as part of the nuclear genome, thus explaining the discrepancy in sizes.

### Gene annotation revealed 3,614 new gene models

Gene annotation of the V4 assembly using MAKER resulted in 15,118 gene models. Compared to the V1 assembly and annotation, which has 12,006 genes, we found 11,504 gene models in common between both versions, while 502 genes are no longer in the new annotation and 3,614 new genes are annotated in V4. Of the 3,614 new gene models, 92.5% have evidence at the transcript level (overlapping RNA-Seq sequences) and 50.0% have predicted functional domains by InterProScan. These values are in accordance with the rest of the gene models in the annotation, which have 92.0 and 59.5% transcript and InterPro domain evidence, respectively. Of the 15,118 gene models, 1,474 proteins show the presence of a signal peptide, based on analysis with SignalP, and 521 are predicted to encode effectors based on analysis with EffectorP (Figure 1). Regarding the mitochondrial genome, V1 contains a total of 41 genes, which includes tRNA, whereas V4 harbors a higher count of 67 genes.

### Synteny analysis between versions shows structural rearrangements

**Figure 2:**
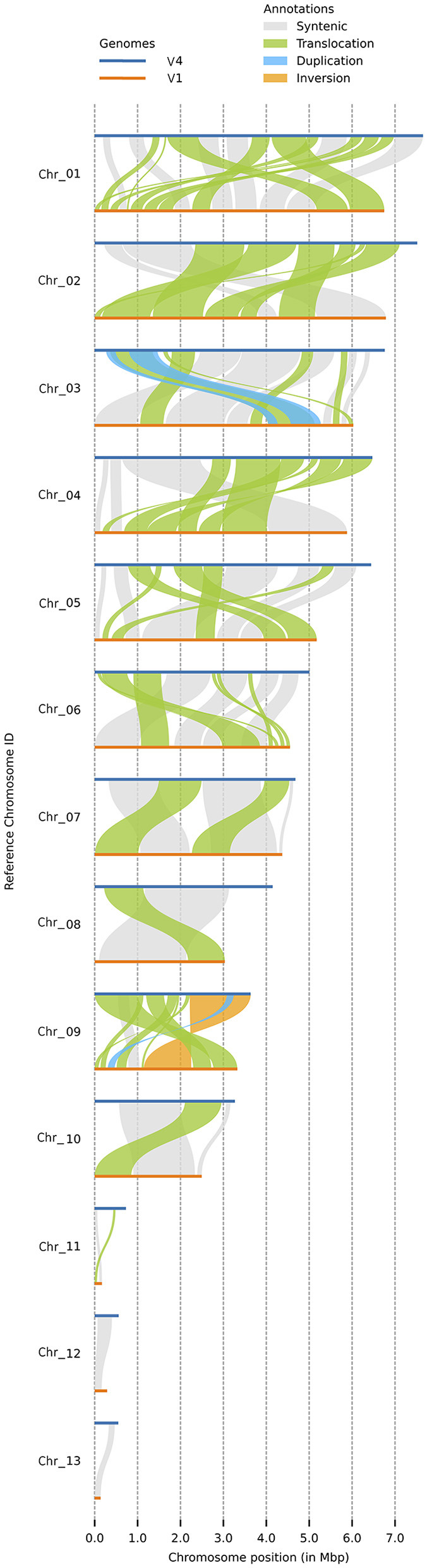
Synteny analysis by plotsr between the two assembly versions. This figure shows the syntenic regions and structural rearrangements with respect to V4. Duplications are present in Chr3 and Chr9, and inversions in Chr9. Regarding the MCs, Chr11 contains one translocation while Chr12 and Chr13 are syntenic. Horizontal blue line: V4 assembly, orange line: V1 assembly.

Both versions of the *C. graminicola* genome were assembled into 13 chromosomes, of which three are MCs (Figure 2). Synteny analysis with plotsr reveals 66 structural rearrangements, of which 61 are translocations, one is an inversion, and four are duplications (Figure 2). Duplications are present in Chr3 and Chr9, and inversions are present in Chr9. Regarding the MCs, Chr11 contains one translocation while Chr12 and Chr13 are syntenic. Altogether, the structural rearrangements represent 23.62 Mb of the genome assembly.

### *C. graminicola* repetitive DNA elements and RIPs found in gene-sparse regions

**Figure 3:**
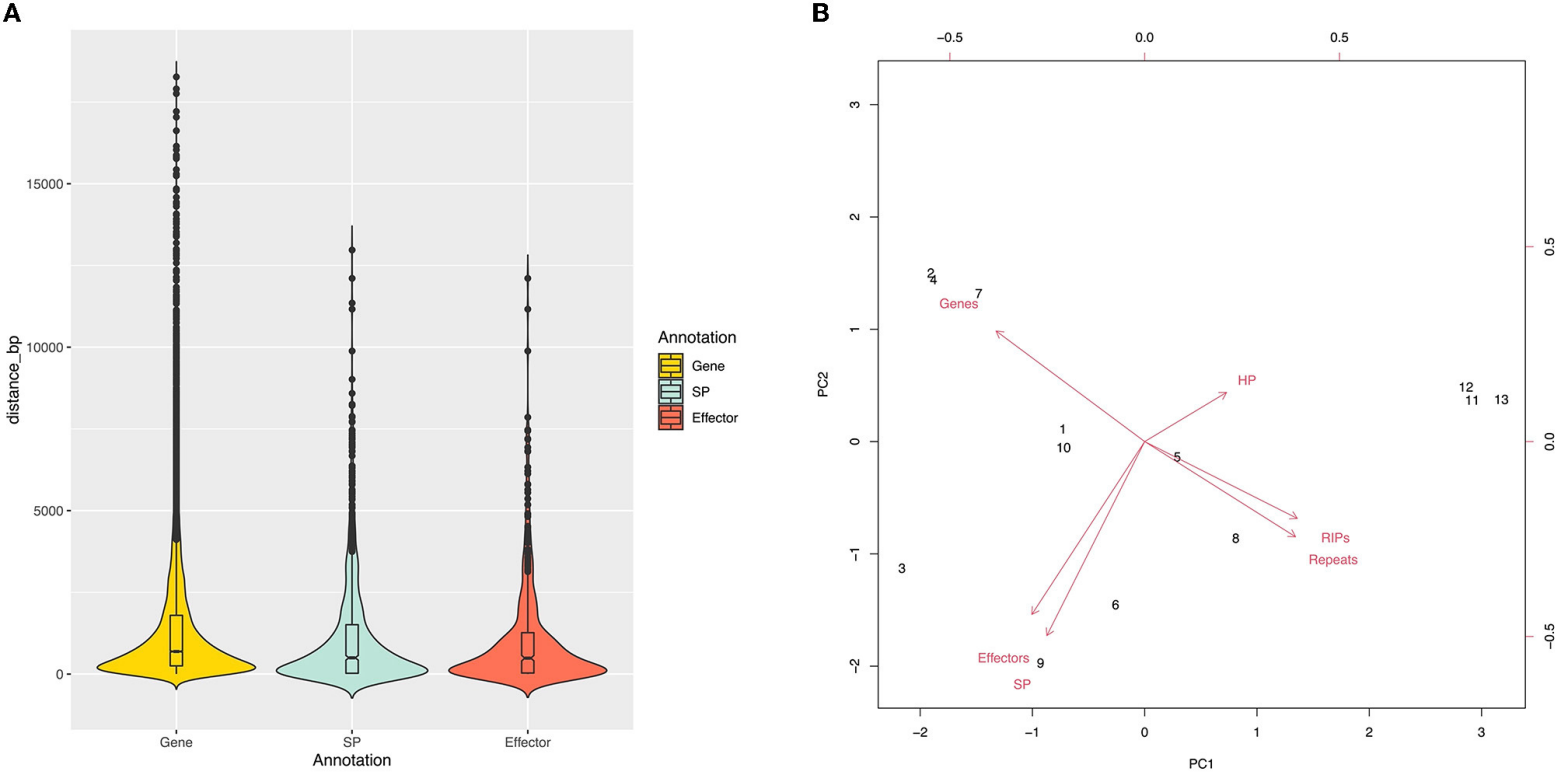
**(A)** Genic distribution compartmentalization. Violin plots represent the intergenic distance of genes and the closest repetitive sequence. **(B)** The core and MCs are grouped by sequence content. Principal component analysis shows two groups, Chr11–Chr13 and Chr1–Chr10.

Approximately 26% of the V4 assembly is comprised of repetitive elements. The region comprising the predicted genes represents a total of 19,862,185 bp, and only 1.33% of these regions have repeats. Of the regions affected by RIP (27.41%), only 0.31% are in the gene region. The density of predicted genes and the repeat content were inversely proportional (Figure 1). We determined the distance between the gene and the nearest repeat was 1,417 bp and the mean distance to secreted proteins (SP) and predicted effectors is less than that of the whole genome, 1,185 and 1,088 bp, respectively, indicating that they tend to be nearer to repeats (Figures 1, 3A). The distance between SP and the nearest repeat was significantly less than genes in the whole genome (*p* = 9.999e-05), as was the distance between effectors and their nearest repeats (*p* = 9.999e-05).

### Minichromosomes lack genes related to pathogenicity

The chromosomes were separated into two groups based on size and gene content (Figure 3B). The first group, comprised of Chr1 to Chr10, is referred to as the “core” chromosomes, while the second group is formed by the MCs, and includes Chr11, Chr12, and Chr13.

When compared to the core chromosomes, the MCs present an enrichment in repeat elements and smaller gene content. The MCs identified in V4 have a higher content of hypothetical proteins when compared to the other chromosomes. However, none of these were predicted as candidate effectors and are completely void of secreted proteins (Table 4). The density of predicted genes per 1 Mb ranged from 243 to 316 in the core chromosomes, while in the MCs, it was 40.

**Table 4:** Differences between core-chromosomes (Chr1–Chr10) and MCs (Chr11–Chr13).

**Characteristic**	**Chr1 to Chr10 (mean)**	**Chr11**	**Chr12**	**Chr13**
Total length (bp)	5,558,768	729,294	559,114	550,543
Number of protein-coding genes	1,500	43	43	35
Proportion of genes by length (%)	35.11	6.29	6.94	5.94
Number of repetitive DNA elements	3,416	706	423	524
Proportion of repetitive DNA elements by length (%)	26.04	68.53	71.87	66.49
GC content (%)	46.30	29.90	30.10	30.30
Proportion of RIPs by length (%)	26.40	76.17	66.54	77.57
Proportion of genes with unknown function (%)	36.51	82.95	81.38	87.09
Proportion of secreted protein encoding genes (%)	2.61	0	0	0
Proportion of effector genes (%)	0.73	0	0	0

The similarity among the 121 genes annotated on the MCs was checked manually. The MC Chr11 shares only 2% of the gene content with other *C. graminicola* strains, while Chr12 and Chr13 share 58 and 57%, respectively, suggesting that Chr11 is dispensable (Table 5). The core group comprises 89 and 94% of the total predicted genes that have biological evidence supported by the presence of transcripts (genes with RNAseq evidence), whereas in the MCs Chr11 has 65%, Chr12 58%, and Chr13 43%. *C. gloeosporioides* (He et al., 1998) and *C. higginsianum* (Plaumann et al., 2018) genomes have been found to include a repeat-rich core and MCs.

**Table 5:** Gene content per chromosome.

**Chromosome**	**Predicted genes**	Orthogroup gene counts^*^	**Genes with RNAseq evidence (%)**	**Core genes (%)**	**Dispensable (%)**	**Repetitive DNA elements (%)**	**RIPs (%)**
Chr1^a^	1,983	1,219	93	61	39	27.52	29.01
Chr2^a^	2,271	1,407	92	62	38	20.98	20.94
Chr3^a^	1,884	1,144	92	61	39	22.27	23.31
Chr4^a^	1,950	1,138	92	58	42	19.64	19.10
Chr5^a^	1,591	1,003	94	63	37	29.20	30.72
Chr6^a^	1,227	762	93	62	38	29.81	30.39
Chr7^a^	1,324	807	93	61	39	21.45	20.44
Chr8^a^	978	618	91	63	37	35.52	35.04
Chr9^a^	911	559	90	61	39	28.86	28.73
Chr10^a^	878	510	89	58	42	25.19	26.33
Chr11^b^	43	1	65	2	98	68.53	76.17
Chr12^b^	43	25	58	58	42	71.87	66.54
Chr13^b^	35	20	43	57	43	66.49	77.57

* Orthogroups present in 21 strains of *C. graminicola*.

a Core chromosomes.

b Minichrosomomes.

### No correlation observed between phylogeny and genome characteristics

**Figure 4:**
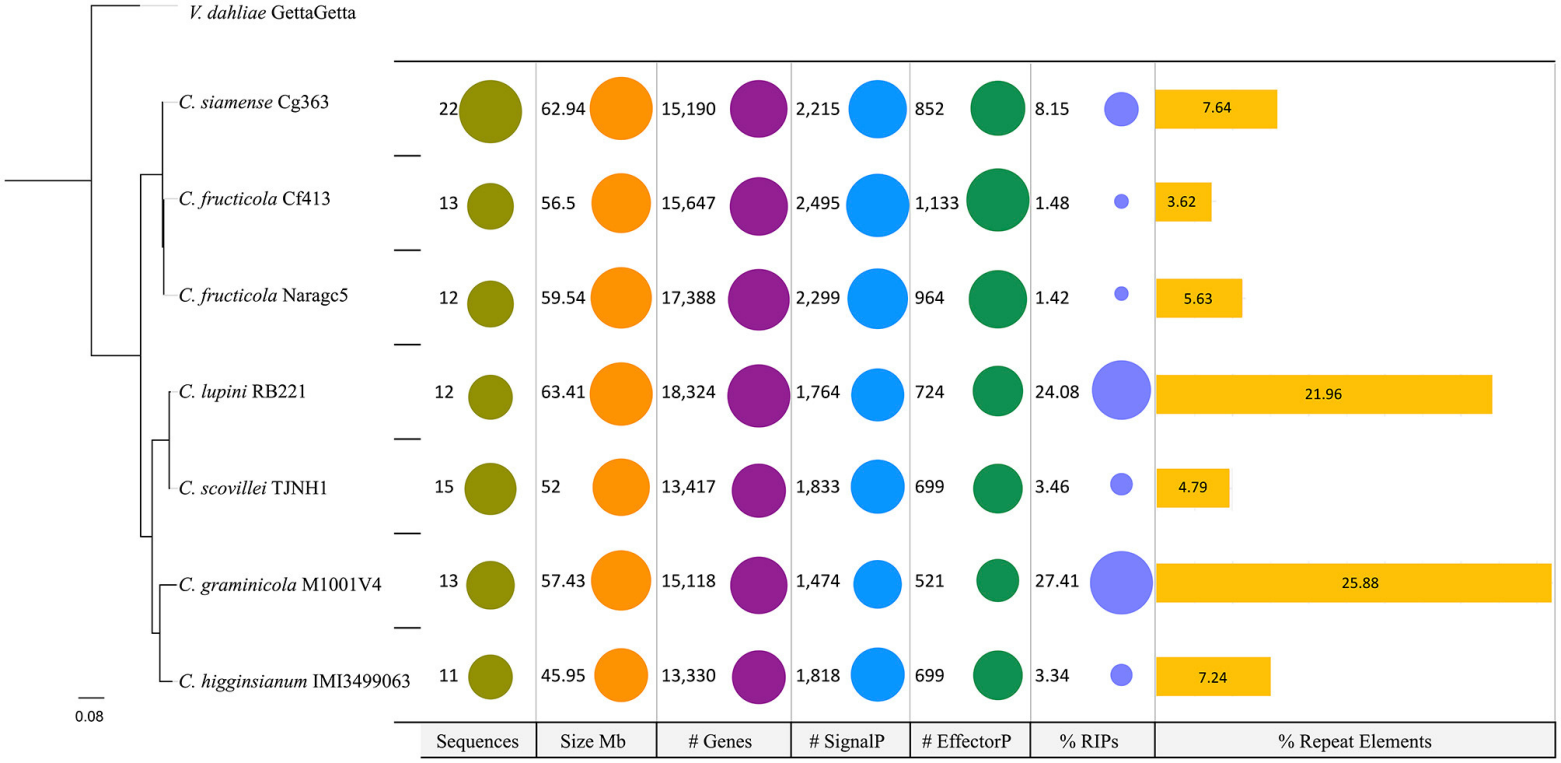
Phylogeny and genomic properties of seven species of the genus *Colletotrichum*. *C. graminicola* and *C. higginsianum* belong to different species complexes; however, the content of repetitive DNA elements differentiates them. *C. lupini* and *C. scovillei* belong to the complex Acutatum, for which these species are observed in the same clade; however, their content of repetitive elements is different. *Verticillium dahliae* was used as the outgroup.

The phylogenomic analysis revealed that closely related species (Table 1) can differ in the number of repetitive DNA elements. *C. lupini* and *C. scovillei* (Figure 4), part of the Acutatum s.c., differ in the percentage of repetitive elements, being 21.96 and 4.79% for *C. lupini* and *C. scovillei*, respectively. *C. graminicola* has 25.88% and *C. higginsianum* 7.24%, and although these do not belong to the same species complex, they are closer species compared to the others included in this analysis.

## Discussion

The evolution of sequencing technologies over the past 10 years, when the first version of the *C. graminicola* genome was published by O'Connell et al. (2012), has allowed the improvement of the assembly of this important pathogen of maize. We used PacBio sequencing to generate 7,331,198 reads (138 Gb), which were assembled into 22 contigs. Short reads from the Illumina sequencing platform were used to generate 87,754,330 reads, which were used to polish the initial assembly. After removing bacterial contamination and the mitochondrial genome for separate analysis, the assembly was comprised of 18 contigs, which were combined into 13 chromosomes after synteny analysis with the previous version of the genome. This led to the identification of three contigs that could be classified as MCs due to their size and similarity to the MCs reported in V1.

Interestingly, synteny analysis between the V4 and V1 assemblies revealed 66 structural rearrangements, including 61 translocations, one inversion, and four duplications. One possible hypothesis to explain these structural rearrangements is that either the V1 or the V4 assembly contains errors. Another possibility is that structural changes in the genome have taken place during the more than 10 years since the sequencing of the V1 assembly, although the cultures are stored at −80°C and steps are routinely taken to limit the number of subcultures.

The new assembly is considerably improved as compared to the previous version (O'Connell et al., 2012), with a higher value of completeness of 98.2%. Other *Colletotrichum* spp. presented annotation completeness ranging from 92.6% for *C. spaethianum* (Utami and Hiruma, 2022) to 97% for *C. higginsianum* (Dallery et al., 2017). In the previous version, no chromosomes were completely assembled, while in the new version, eight core chromosomes, and two MCs were completely assembled. Chr10 comprises rDNA grouping at the 3′ end in our V4 assembly, for which we have identified this large region as a NOR (nucleolar organizing region). Taga et al. (2015) determined the cytological karyotype of *C. graminicola* M1.001, where a thread-like protrusion and different colors were observed, for which the authors associate this region on Chr10 with the NOR-Chr. These results agree with our assembly in the same chromosome (Chr10) by identifying rDNA in our nuclear genome. In most eukaryotes, this NOR region exists for one or more chromosomes (Gregory, 2005), and the presence of this NOR-Chr seems to be a feature of fungi displayed as a long protrusion (Taga et al., 2015). The lack of a telomere in Chr10 could be attributed to the rDNA content (Wu et al., 2009), although we cannot rule out the possibility that the missing telomeres are due to incomplete sequencing. The mitochondrial genome has a GC content of 29.6%, similar to that of the previous assembly (29.9%) (O'Connell et al., 2012), *C. acutatum* (30.10%) (Baroncelli et al., 2014), *C. lupini* 29.90% (Baroncelli et al., 2021), and *C. siamense* (35.45%) (Cho et al., 2022).

Gene annotation of the V4 assembly revealed 15,118 genes, with 3,614 of them being new predictions compared to V1. A total of 11,504 genes were common to both versions, while 502 genes were no longer present in the new annotation. Among the new gene predictions, 1,474 were secreted proteins, 521 of which were predicted as effectors. We found that 26% of the V4 assembly was made up of repetitive elements, with the gene-sparse regions being particularly enriched in repeats. We also identified RIPs in the genome, with a higher density of RIPs in the MCs compared to the “core” chromosomes (Chr1–Chr10). The MCs were found to be enriched in repeats and to have reduced gene content compared to the core chromosomes, with no predicted secreted protein-coding genes and more hypothetical genes. We also found that secreted proteins and predicted effectors tend to be located closer to repeats than the rest of the genome. The characterization of repeat-rich regions paves the way for this higher-resolution assembly, as these regions are often the most difficult to assemble (Treangen and Salzberg, 2012). We found a higher percentage of repetitive elements in V4 when compared to V1, indicating improved assembly of these regions. The new assembly will also aid in the identification and characterization of genes encoding proteins with tandem repeats that we have found to be incorrectly annotated in the V1 assembly (data not shown) (Vargas et al., 2016). Previous results of *C. graminicola* strains assembled only with short-reads have shown that repetitive sequences are not adequately assembled (Crouch et al., 2014). This study confirmed that secreted proteins and effector candidates are found mostly near regions rich in repetitive DNA elements, similar to *C. higginsianum* (Dallery et al., 2017).

The genome of strain M1.001 (V4) has three assembled sequences of MCs, two of which have both telomeric regions. More than 80% of the 121 genes found in MCs are defined as hypothetical proteins, and none of them are related to pathogenicity genes, including candidate effector encoding genes. Orthogroups analysis and manual verification showed that only Chr11 may be dispensable because only one of its genes was present in other strains. More than 50% of all the genes on Chr12 and Chr13 have predicted orthologs in other strains, suggesting that they are conserved. For this reason, we cannot suggest that these two are dispensable chromosomes as Bertazzoni et al. (2018) also suggested that minichromosomes are not necessarily accessory (dispensable) chromosomes.

The influence of RIP is higher in MCs when compared to core chromosomes, a feature that is common in the MCs of plant pathogenic fungi (Peng et al., 2019; Langner et al., 2021). These MCs contain AT-rich blocks and play an important role as a defense mechanism to protect against the proliferation of TEs (Rouxel et al., 2011; Fouché et al., 2022). These differences are also shown in the percentage of RIPs. It has previously been described that the plasticity and architecture of plant pathogen genomes result from significant variations in the size and content of repetitive DNA elements (Möller and Stukenbrock, 2017; Lorrain et al., 2021). The MCs are enriched in repeat elements and have smaller gene content. The same result was found in *M. oryzae* (Langner et al., 2021). Unlike other studies (Bhadauria et al., 2019), we have not found that the genes present in MCs are involved in virulence or encode effector candidates. We wanted to verify if this same behavior exists in the entire genus of *Colletotrichum*, but unfortunately, no assemblies at the chromosome level are available that allow us to delve a little deeper into the importance of minichromosomes, being a subject that is still poorly understood.

Genomic compartmentalization has been reported in other fungal pathogens (Croll and McDonald, 2012; Raffaele and Kamoun, 2012; Derbyshire et al., 2017; Möller and Stukenbrock, 2017; Tsushima et al., 2019) and is associated with genomic plasticity hypervariable genomic regions found in these fungi. Although we observe a clear enrichment of proteins of unknown function in the MCs, as was found in *Magnaporthe oryzae* by Langner et al. (2021), it is not clear that having a large number of this type of protein involves them with any particular functions.

Comparative genomics with other species of *Colletotrichum* revealed no association between phylogeny and other genomic features such as genome size or repetitive DNA content. *Colletotrichum lupini* and *C. scovillei*, both of which belong to the same species complex, have large differences in repetitive element content. The same occurs for *C. graminicola* and *C. higginsianum*, which, although they are not from the same complex of species, show clear differences in repeat content.

We wanted to verify if this same behavior exists in the entire genus *of C. graminicola*, but unfortunately, there are no chromosome-level assemblies that allow us to delve deeper into the importance of MCs. The biology of MCs is a subject that is still poorly understood, although, in recent years, complete MC sequences were also reported in the genome assemblies of other plant pathogenic fungi (Baroncelli et al., 2021; Gan et al., 2021; Langner et al., 2021; Zaccaron et al., 2022).

In conclusion, we have improved the genome assembly of *C. graminicola*, revealing new insights into the structure and content of the genome. We have identified structural rearrangements and RIPs, and have found that the MCs are enriched in repeats and have a reduced gene content compared to the core chromosomes. We also found that secreted proteins and predicted effectors tend to be located closer to repeats than the rest of the genome. This study sheds light on important aspects of the architecture and organization of the *C. graminicola* genome, which will be an important source for future studies of the genus *Colletotrichum* and the evolution of *C. graminicola*.

## Data availability statement

The datasets presented in this study can be found in online repositories. The names of the repository/repositories and accession number(s) can be found below: https://www.ncbi.nlm.nih.gov/genbank/, PRJNA900520.

## Author contributions

SB, RB, and MT conceived and designed the experiments, performed the bioinformatic analyses, and wrote the manuscript. SB, RB, TB, SS, and MT contributed intellectually. All authors read, revised, and agreed with the final version of the manuscript.
